# Influence of Alloying Element Mg on Na and Sr Modifying Al-7Si Hypoeutectic Alloy

**DOI:** 10.3390/ma15041537

**Published:** 2022-02-18

**Authors:** Chunfa Huang, Zhiguo Liu, Jianguo Li

**Affiliations:** 1 General Research Institute for Nonferrous Metals, Beijing 100088, China; huangchunfa2012@163.com (C.H.); zhgliu@grinm.com (Z.L.); 2 GRIMAT Engineering Institute Co., Ltd., Beijing 101407, China; 3 State Key Laboratory of Advanced Materials for Smart Sensing, GRINM Group Co., Ltd., Beijing 100088, China; 4 Laboratory of Advanced Materials, School of Materials Science and Engineering, Tsinghua University, Bejing 100084, China

**Keywords:** alloying element magnesium, Al-7Si-0.4Mg alloy, Na modification, Sr modification, modification efficiency

## Abstract

The influence of alloying element Mg on Na and Sr modifying Al-7Si hypoeutectic alloys was investigated. The residual content of Na and the morphology of modified eutectic silicon were characterized. It was found that the alloying element Mg had an enhanced effect on the uptake of sodium in the Al-7Si hypoeutectic alloy modified by the Na-contained modifier. Moreover, the morphology of eutectic silicon of the modified Al-7Si alloys was significantly different from that of Al-7Si-0.4Mg alloys in the present research. When the addition of the modifier is enough, both modifiers could entirely modify the eutectic silicon phase of Al-7Si alloys, while incompletely modified eutectic silicon was observed in both Na-modified and Sr-modified Al-7Si-0.4Mg alloy. It was observed that there was an adhering relationship between the partially modified eutectic silicon with Mg-rich phases. According to the results, it can be proposed that the addition of Mg will affect the solidification behavior of alloys, thereby, leading to the incomplete modification of eutectic silicon phases.

## 1. Introduction

Al-Si hypoeutectic alloys are extensively used in aerospace, electronic communication and automobile manufacturing, owing to their excellent casting performance, fatigue resistance and corrosion resistance [1]. The Al-Si system is a simple binary eutectic with limited solubility of Al in Si and limited solubility of Si in Al. The mechanical properties of hypoeutectic Al-Si alloy are mainly determined by the morphology, size and distribution of primary α-Al phases, eutectic silicon phases, intermetallic compounds, and defects. The morphology, size and distribution of eutectic silicon have prominent influence upon alloy properties [2]. The morphology of unmodified eutectic silicon is coarse and lamellar. The eutectic silicon is a brittle phase. The unmodified eutectic silicon provides a potential site for the formation of a cracking during plastic deformation, which will result in a significant decrease in mechanical properties of the alloy [3,4]. Modification is the most effective way to improve the ductility and toughness of Al-Si alloy [5]. The morphology of eutectic silicon can be transformed from flake-like to fibrous by modification treatment. The structure of eutectic silicon can be controlled by the addition of certain modifier elements such as Na [5], Sr [6], Sb [7], Eu [8], Ca [9], Yb [10], among which Na and Sr are the most widely used in the aluminum industry. Sodium was the first modifying element extensively studied. It is usually added to the molten aluminum in the form of a mixture of salts containing NaF. Only a trace amount (0.005 wt.%~0.01 wt.%) of Na is effective to achieve the full modification of eutectic silicon [11]. Due to the excellent modification performance and low cost, it is widely used in industries. At present, however, Sr has gradually replaced Na as a modifier for cast Al-Si alloys, because of the advantages of zero-emissions and good remelting properties. Complete modification of the eutectic Si phase could be achieved with 0.03 wt.% Sr [12,13].

A356 alloy is a magnesium-containing cast Al-7Si alloy that is widely used in automobile industry, for engine components, wheels and cylinder liners, etc. [14,15,16] The Mg content of the commercial A356 alloys ranges from 0.25 to 0.45 wt.%. Mg is usually introduced to induce an age-hardening effect through the precipitation of Mg_2_Si particles [17]. The addition of Mg has a beneficial influence on the eutectic silicon in unmodified Al-Si alloys; it could refine and modify the eutectic silicon in Al-Si hypoeutectic alloy [18,19,20,21,22]. However, some studies in the literature reported that the addition of Mg has a negative effect on the modification of eutectic silicon. Joenoes et al. [23] reported that the alloying element Mg posed a negative effect on the Sr modification, which changes in the microstructure from well modified to partially modified. The reason for the degradation of modification was attributed to the formation of (Mg_2_SrAl_4_Si_3_) compounds. Zhang et al. [24] suggested that the partially modified eutectic silicon was related to the formation of π(Al_8_FeMg_3_Si_6_) phase in Sr-modified A356 alloy. Chacko et al. [25] suggested that the modification efficiency of Sr deteriorated with the addition of Mg. Unfortunately, they did not conduct further research on the underlying mechanism. In addition, to the best of our knowledge, the effect of alloying element Mg on the morphology of eutectic silicon modified by Na, which is another widely used modifier, is rarely reported. The influence mechanism of alloying element Mg on the morphology of the modified eutectic silicon has not been systematically studied. The partially modified eutectic silicon makes the eutectic silicon structure heterogeneous, which is detrimental for the properties of the alloys and hinders its further development. The lack of understanding of the fundamental mechanism underlying eutectic modification impedes further improvement of these alloys. It is necessary, therefore, to clarify the influence of Mg on Si modification in the presence of Na and Sr and the relationship between the Mg-rich phase and partially modified eutectic silicon, based on solid experimental evidence.

In the present research, the authors attempted to investigate the influence of the alloying element Mg on the morphology of eutectic silicon in modified Al-Si hypoeutectic alloy. The effect of Mg on the residual Na content in Al-Si alloy modified by Na-contained salt was examined in this paper. The morphology of eutectic silicon was characterized. The effect of the alloying element Mg on the Na-based modification and Sr-based modification of Al-Si alloy was discussed. Based on the results, the related mechanism was further proposed. It could provide a more accurately theoretical basis for understanding and mastering the modification process of Al-Si alloy.

## 2. Materials and Methods

Two groups of alloys were employed in this experiment. The first group of alloys was prepared with commercial pure materials, and the second group was prepared with materials in high purity. Commercial pure Al-7Si and Al-7Si-0.4Mg alloys used in the present investigation were prepared by Al (99.7 wt.% in purity), Mg (99.7 wt.% in purity) and Si (99.9 wt.% in purity), which corresponds to the purity usually used on the industrial scale. High pure Al-7Si and Al-7Si-0.4Mg alloys were prepared with 99.99 wt.% Al, 99.99 wt.% Mg and 99.999 wt.% Si. The amount of Na has a great influence on the morphology of eutectic silicon; only a trace amount of Na is effective to achieve the desired modification level. Therefore, it is necessary to accurately determine the Na content in the alloy. The composition of alloys is given in Table 1.

As is shown in Table 1, there are different amounts of sodium in the Al-7Si and Al-7Si-0.4 Mg alloys, which merely differ from Mg content, because the raw Mg material would introduce more Na into the alloys. On the other hand, the different amounts of Na in Al-7Si and Al-7Si-0.4Mg alloy can be considered within the measurement error range.

The Na-based modifier consists of 40 wt.%NaCl, 40 wt.%KCl and 20 wt.%NaF. The mixture of salts has a low eutectic melting point and can react with molten aluminum in a liquid state. These salts were mixed, then melted at 750 °C. After cooling, the modifier was milled into powders. The modifier components used in the experiment were analytically pure.

The pre-prepared Al-7Si and Al-7Si-0.4Mg alloys were melted in a resistance furnace and held at 750 °C. The Na-based modifier and Sr were added for modification treatment. Sr was added to the melt by Al-10Sr master alloy, with the amount of 0.03 wt.% and 0.06 wt.%. The Na-based modifiers were added to the melt at 0.00 wt.%, 0.2 wt.%, 0.4 wt.% and 0.6 wt.%, respectively. The slag formed upon the melt surface was skimmed after holding for 8 min; the alloy melt was subsequently poured into a permanent mold with a cooling rate of 15 K/s.

The samples for analysis were ground and polished in compliance with standard metallography techniques. The chemical composition of the alloys was analyzed by SPECTROMAXx spark spectrometer (Spectro, Kleve, Germany). The detection accuracy of the spark spectrometer proofread by standard sample can reach 1 ppm, which was required for our work, because the modification level is very sensitive to the amount of Na. The samples used for microstructural characterization were electrochemically polished. The electrolytic polishing solution is made by mixing perchloric acid with anhydrous ethanol in a ratio of 1:9. A HITACHI SU8220 cold field emission scanning electron microscope (SEM) integrated with a flat-Quad EDX (Hitachi High-Tech Corporation, Tokyo, Japan) was used to analyze the morphology of the eutectic silicon and the intermetallic phases composition.

## 3. Results and Discussions

**Figure 1:**
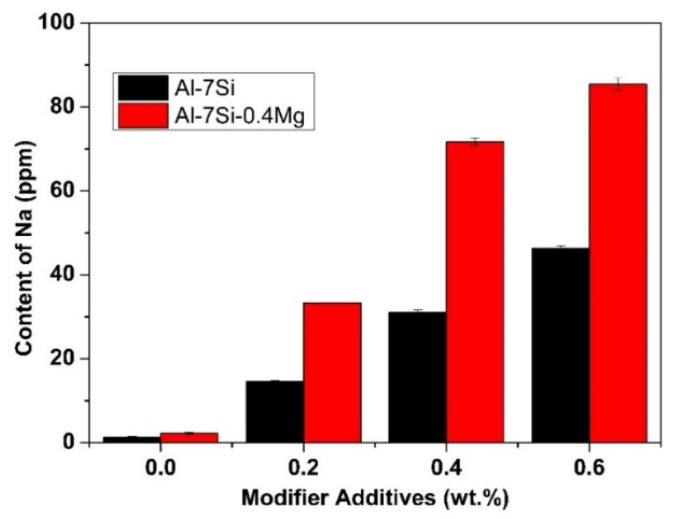
Effect of the addition of Na-based modifier on sodium content in commercial pure Al-7Si and Al-7Si-0.4Mg alloys.

The sodium element was introduced into the alloy through the reactions between NaF in the sodium-contained salt modifier and the alloy melt: Al + 3NaF → 3Na + AlF_3_, Mg + 2NaF → 2Na + MgF_2_. The modification efficiency of Al-Si alloy is related to the residual amounts of sodium, which depends on the addition rate of the Na-based modifier. The effect of modifier addition amount on the residual sodium content is shown in Figure 1.

As can be seen from Figure 1, the residual amount of sodium in commercial pure Al-7Si-0.4Mg alloy was significantly higher than that of commercial pure Al-7Si alloy at the same addition of Na-modifier. The initial sodium content of commercial pure Al-7Si and Al-7Si-0.4Mg alloy was 1.3 ppm and 2.2 ppm, respectively. After adding 0.2 wt.%, 0.4 wt.%, 0.6 wt.% of Na-modifier, residual sodium levels increased to 14.6 ppm, 31.1 ppm, 46.3 ppm and 33.3 ppm, 71.7 ppm, 85.4 ppm, respectively. The equilibrium constants of the substitution reaction between NaF and Al, Mg at 750 °C [26] were 6.95 × 10^−9^ and 3.59 respectively. Sodium was derived from the reaction between NaF and Al in Al-7Si alloy. Whilst, in Al-7Si-0.4Mg, sodium was introduced by a thermodynamically more favorable reaction, i.e., the one between NaF and Mg. The equilibrium constants of the reaction between NaF and Mg are much higher than that between NaF and Al. Therefore, more sodium was picked up in Al-7Si-0.4Mg than Al-7Si alloy at the same addition of Na-based modifier.

**Figure 2:**
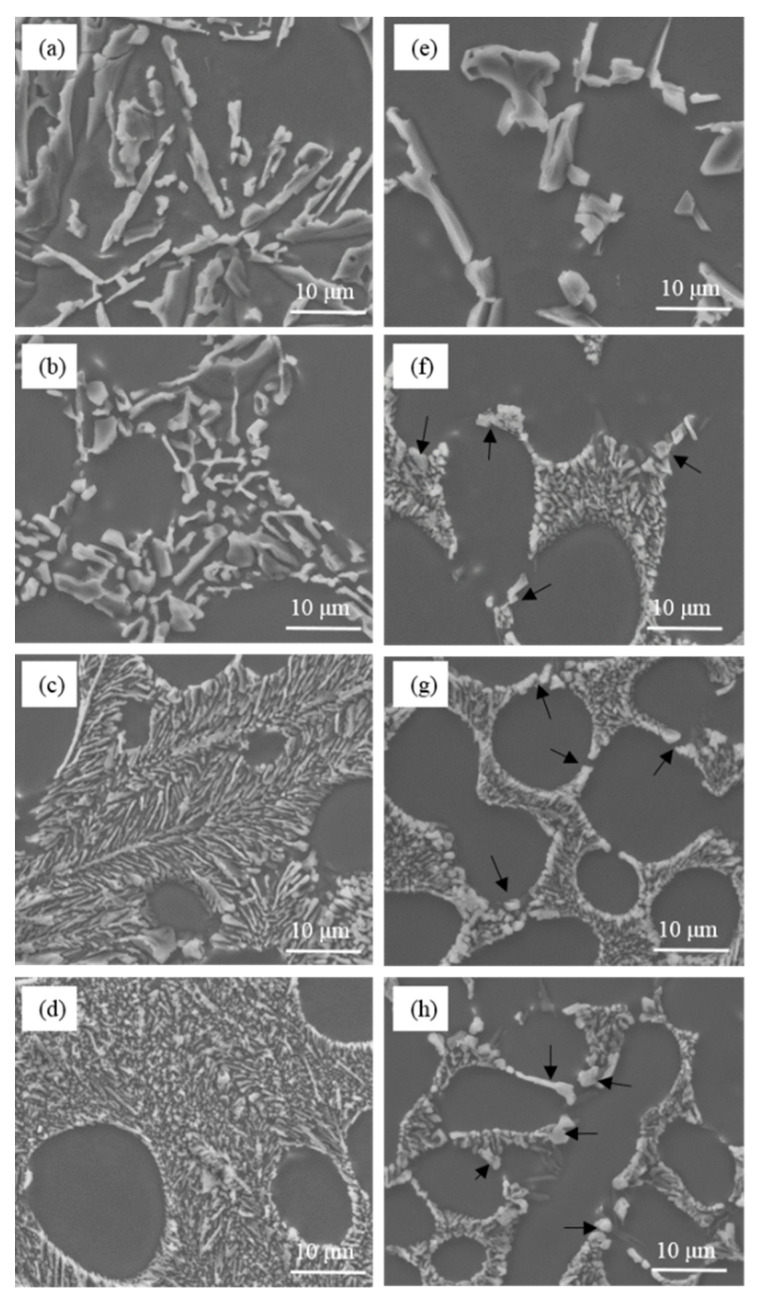
Microstructure of commercial pure Al-7Si alloy and Al-7Si-0.4Mg alloy with different Na-based modifier addition. (**a**–**d**) Al-7Si alloy; (**e**–**h**) Al-7Si-0.4Mg alloy; (**a**,**e**) without modifier; (**b**,**f**) with 0.2 wt.% modifier; (**c**,**g**) with 0.4 wt.% modifier; (**d**,**h**) with 0.6 wt.% modifier.

As is shown in Figure 2, the unmodified eutectic silicon in commercial pure Al-7Si and Al-7Si-0.4Mg alloys adopted a flake-like structure. The morphology of eutectic silicon modified showed a significant difference between commercial pure Al-7Si and Al-7Si-0.4Mg alloys. The content of Sr in alloys presented in Figure 2 remains in an extremely low level (2 ppm), which can be considered as an impurity element that does not pose an effect upon microstructure. The morphology of eutectic silicon only depends on the amount of Na. When 0.2 wt.% of Na-based modifier was added, the eutectic silicon in Al-7Si alloy shows a refined structure. In Al-7Si-0.4Mg alloy, the eutectic silicon morphology was fibrous at the core of the eutectic structure; some coarse eutectic silicon could be observed at the edge of the eutectic structure. When the addition increased to 0.4 wt.%, the eutectic silicon phases in the Al-7Si alloy were transformed into fibrous morphology; only a small amount of eutectic silicon remained partially modified. The eutectic silicon in Al-7Si alloy was fully modified when the addition of modifier was added up to 0.6 wt.%. It is noteworthy that incompletely modified, massive eutectic Si with irregular shapes could be observed in the Al-7Si-0.4Mg alloy with a higher addition of Na-based modifier (as shown by the black arrows in Figure 2f–h).

As can be seen from Table 1, the difference between commercial purity Al-7Si and Al-7Si-0.4Mg alloy was merely Mg content, except the tiny difference in impurity content. However, the morphology of modified eutectic silicon showed a significant difference. A better modification efficiency can be obtained by increasing the content of sodium in Al-7Si alloy. When the Na content reached 46.3 ppm, the eutectic silicon of Al-7Si alloy underwent the desired modification. With the addition of 0.2 wt.% of Na-based modifier, due to the presence of alloying element Mg, more sodium was introduced into Al-7Si-0.4Mg alloy (as shown in Figure 1); the modification efficiency was better than that of Al-7Si alloy. On the other hand, Al-7Si-0.4Mg alloy always exhibited incomplete modification, even after increasing the Na content. Therefore, it can be inferred that the alloying element Mg had a weakening effect on the modification of eutectic silicon when the Na content was enough for modification.

**Figure 3:**
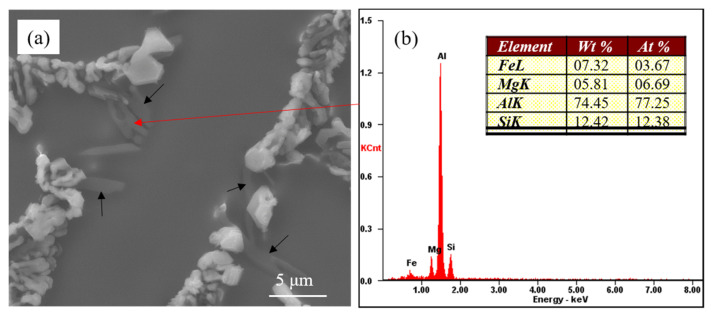
(**a**) Morphology of partially modified eutectic Si and Mg-rich phase in commercial pure Al-7Si-0.4Mg alloy; (**b**) Corresponding EDS results.

**Figure 4:**
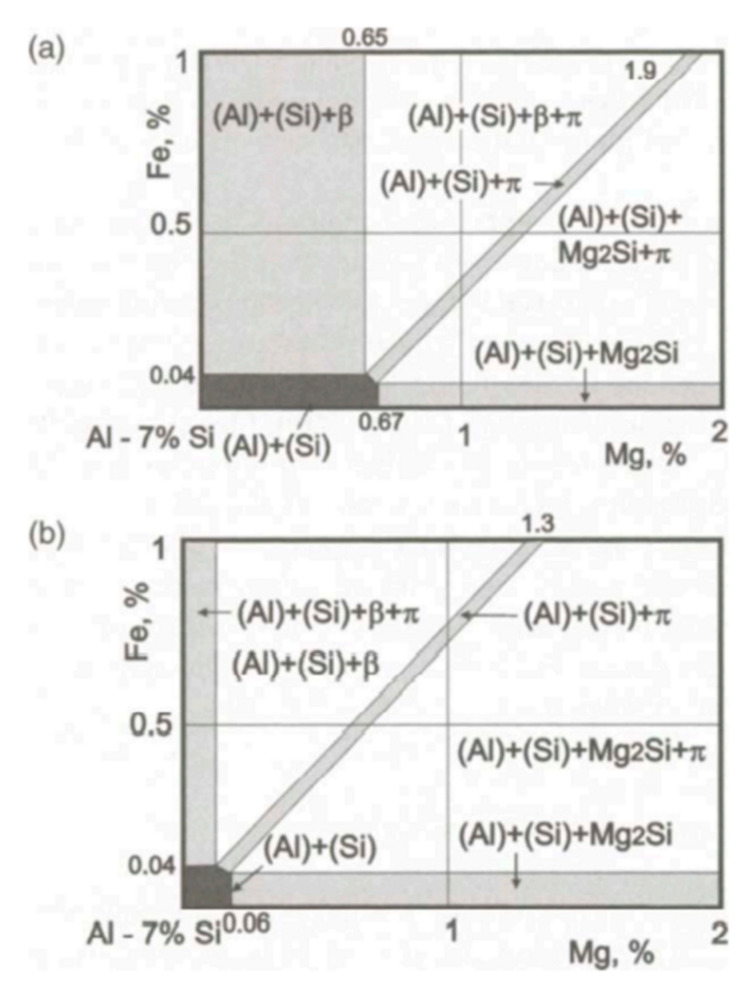
Isothermal sections of Al-Fe-Mg-Si phase diagram at 7% Si: (**a**) 540 °C and (**b**) 200 °C; β-Al_5_FeSi, and π-Al_8_FeMg_3_Si_6_ [27].

Figure 3 presents the partially modified microstructure of commercial pure Al-7Si-0.4Mg alloy with 0.6 wt.% Na-based modifier. As can be seen, the eutectic silicon phases adhering to the Mg-rich phases (as shown by the black arrow in Figure 3a) were coarse, exhibiting incomplete modification features. The eutectic silicon at the core of eutectic structure where the Mg-rich phases did not display showed a coral-like structure. It can be inferred that the presence of the coarse eutectic silicon in the modified Al-7Si-0.4Mg alloy had a close relationship with the Mg-rich phases. The EDS of Mg-rich phases revealed that these phases contained Al, Fe, Mg and Si (as shown in Figure 3b). According to the equilibrium phase diagram [27] (as shown in Figure 4), at low magnesium concentrations and a typical Fe impurity level (A356 type alloys), Fe will be bound in the β(AlFeSi) phases. However, under real, nonequilibrium conditions, solidification is completed by the invariant eutectic reaction: L → (Al) + (Si) + Mg_2_Si + π(Al_8_FeMg_3_Si_6_) at 554 °C. The β(AlFeSi) phase is completely replaced by the quaternary compound that binds almost all iron [17]. According to the equilibrium phase diagram [27], there should be α-Al phases, eutectic silicon phases, Mg_2_Si phases and π(Al_8_FeMg_3_Si_6_) phases in the Al-7Si-0.4Mg alloys. When we analyzed the microstructure of samples through SEM, we tried to convert multiple fields of view, Unfortunately, we could not find the Mg_2_Si phase; only the π(Al_8_FeMg_3_Si_6_) phase was observed, which presented at the edge of incompletely modified eutectic silicon. Given the EDS of the Mg-rich phase, it can be speculated that the Mg-rich phases in Figure 3 were π(Al_8_FeMg_3_Si_6_). As mentioned above, the presence of coarse eutectic silicon in the modified Al-7Si-0.4Mg alloy correlated with the π(Al_8_FeMg_3_Si_6_).

**Figure 5:**
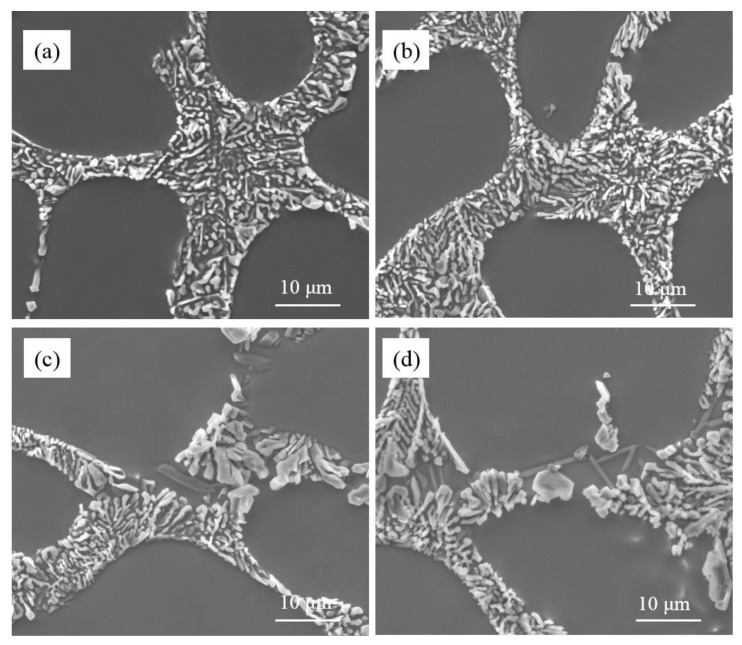
Microstructure of commercial pure Al-7Si alloy and Al-7Si-0.4Mg alloy with different Sr additions; (**a**,**b**) Al-7Si alloy; (**c**,**d**) Al-7Si-0.4Mg alloy; (**a**,**c**) with 0.03 wt.% Sr; (**b**,**d**) with 0.06 wt.% Sr.

Figure 5 shows the morphology of the eutectic Si phase in the commercial pure Al-7Si alloy and Al-7Si-0.4Mg alloy modified by Sr. The initial amount of Na in the alloys is very small, which is inevitably present in alloys, and is considered an impurity element, which would not affect the microstructure. With the same addition of Sr, the morphology of eutectic silicon was different between the commercial pure Al-7Si alloy and Al-7Si-0.4Mg alloy. When 0.03 wt.% of Sr was added, the eutectic silicon presented a fibrous morphology in commercial pure Al-7Si alloy, whilst coarse eutectic silicon with irregular shapes was observed in commercial pure Al-7Si-0.4Mg alloy. As the Sr addition increased to 0.06 wt.%, the fibrous eutectic silicon phase became more refined in the Al-7Si alloy, while the eutectic silicon phase remained partially modified in the Al-7Si-0.4Mg alloy. The eutectic silicon of Al-7Si alloy underwent the desired modification (see Figure 4a,b), while the eutectic silicon of Al-7Si-0.4Mg alloy exhibited incomplete modification. It can be inferred that alloying element Mg does indeed impair Sr modification.

**Figure 6:**
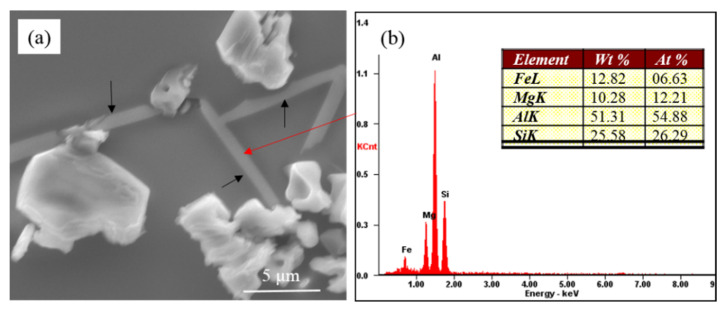
(**a**) Morphology of partially modified eutectic Si and Mg-rich phase in commercial pure Al-7Si-0.4Mg alloy; (**b**) Corresponding EDS results.

Figure 6 presents the morphology of partially modified eutectic Si and Mg-rich phase in commercial pure Al-7Si-0.4Mg alloy with 0.06 wt.% Sr. Similar to the Na-modified Al-7Si-0.4Mg alloy, the partially-modified eutectic silicon had close contact with the Mg-rich phases in Sr-modified Al-7Si-0.4Mg (marked by the black arrow in Figure 6a). According to the corresponding EDS result, the Mg-rich phase in Sr-modified Al-7Si-0.4Mg is the same phase as that in Na-modified Al-7Si-0.4Mg, which is the π(Al_8_FeMg_3_Si_6_) phase. Joenoes et al. [23] believed that the formation of the (Mg_2_SrAl_4_Si_3_) compound resulted in a reduction in the amount of effective Sr and a weaking of the modification efficiency of Sr. Unfortunately, so far, no information on the (Mg_2_SrAl_4_Si_3_) compound appears in the literature. In the present study, no Sr was detected in the Mg-rich phase. In addition, the formation of multiple compounds cannot explain the occurrence of incomplete modification of the Na-modified Al-7Si-0.4Mg alloy, as no compounds containing Mg and Na have been found in the reported literature. To the best of our knowledge, the relation of the presence of coarse eutectic silicon and π phase has been rarely reported in the literature. In the Sr-modified A356 alloy, Zhang et al. [24] observed that the eutectic silicon near the π(Al_8_FeMg_3_Si_6_) phases always exhibited incomplete modification. Nevertheless, some partially modified eutectic silicon was also observed in the sites without Fe at the edge of the eutectic structure in their research. They suggested that the incomplete modification was not only due to the presence of Fe. Unfortunately, they did not conduct further in-depth studies to figure out the relationship between the Mg-rich phases and the partially modified eutectic silicon. Based on the present results and those in the literature, it is reasonable to believe that the presence of Mg and Fe is responsible for incomplete modification of the Al-7Si-0.4Mg alloy.

Shankar et al. [28] and Lan et al. [29] reported that there was an adhering relationship between the β(AlFeSi) phase and the partially modified eutectic silicon in the Al-8Si alloy, which was supported by the research of Zhang et al. [30] and Li [31] et al, who observed that eutectic silicon nucleated on the β(AlFeSi) phases. Lan et al. [29] suggested that the incomplete modification phenomenon could be eliminated by reducing the Fe content in the Al-8Si alloy and by using high-purity raw materials.

**Figure 7:**
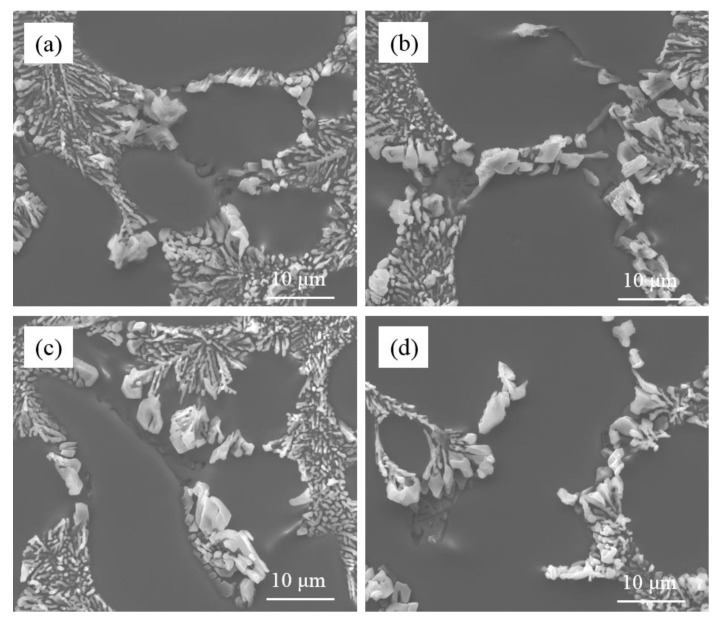
Microstructure of high pure Al-7Si-0.4Mg alloy with different modifier additions; (**a**) with 0.4 wt.% Na-modifier; (**b**) with 0.6 wt.% Na-modifier; (**c**) with 0.03 wt.% Sr; (**d**) with 0.06 wt.% Sr.

In order to eliminate the interference of Fe, the high pure Al-7Si-0.4Mg alloys modified were prepared. Figure 7 presents the microstructure of high pure Al-7Si-0.4Mg alloys modified by Na and Sr. Similar to the commercial pure Al-7Si-0.4Mg alloys, partially modified structure was observed in the modified high pure Al-7Si-0.4Mg alloys of both Na modification and Sr modification. The microstructure of high pure Al-7Si-0.4Mg alloy did not change significantly with the increase of modifier addition. There were always some coarse eutectic silicon phases at the edge of the eutectic structure. Thus, the unmodified state of eutectic silicon was not simply due to the presence of Fe. Reducing the Fe content in the Al-7Si-0.4Mg alloy could not improve the modification efficiency.

**Figure 8:**
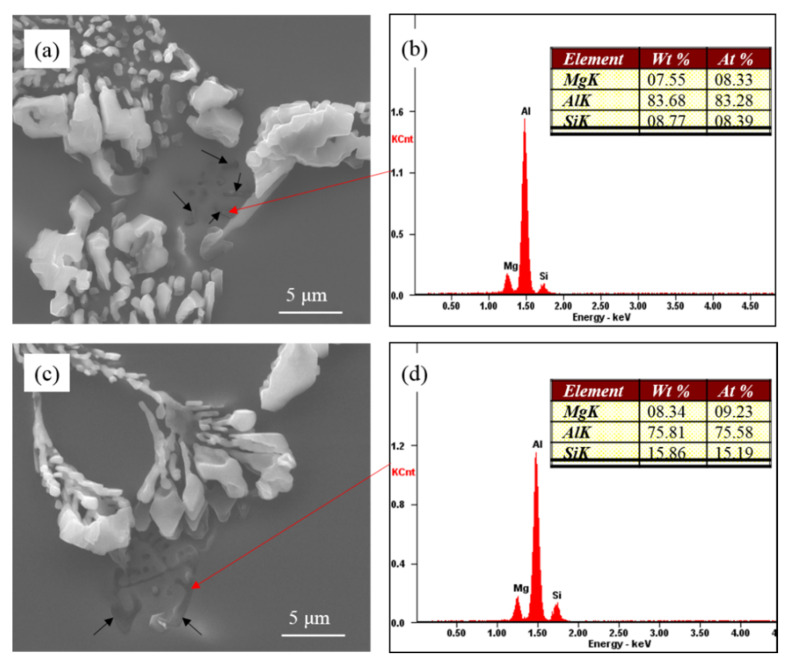
Morphology of partially modified eutectic Si and Mg-rich phase in high pure Al-7Si-0.4Mg alloy; (**a**) modified by Na; (**c**) modified by Sr; (**b**,**d**) Corresponding EDS results.

As shown in Figure 8, there is a close relationship between coarse eutectic silicon and the Mg-rich phase. According to the phase diagram of Al-Si-Mg ternary alloy [27], only α-Al, Si and Mg_2_Si phases are presented in the Al-7Si-0.4Mg alloy. The binary (Al + Si) eutectics always forms in the temperature range of 577 °C~550 °C after the primary crystallization of (Al). The ternary eutectics (Al + Si + Mg_2_Si at 550 °C) forms only as a result of non-equilibrium solidification. According to the EDS of Mg-rich phase, it can be assumed that the alloying element magnesium in the high pure Al-7Si-0.4Mg alloy formed the Mg_2_Si phase during solidification in the present investigation. In Figure 8, the Mg-rich phase is the Mg_2_Si phase.

It becomes clear that the eutectic silicon exhibited incomplete modification in Al-7Si-0.4Mg alloy for both Na modification and Sr modification. There is a close relationship between the partially modified eutectic silicon with Mg-rich phases. In commercial pure Al-7Si-0.4Mg alloy, the coarse eutectic silicon is related to the π(Al_8_FeMg_3_Si_6_) phase. While, in highly pure Al-7Si-0.4Mg, the partially modified eutectic silicon is related to Mg_2_Si phases. Due to the presence of the alloying element Mg, the binary eutectic reaction is transformed into a ternary or quaternary eutectic reaction during the final stage of solidification. According to the phase diagram, both the π(Al_8_FeMg_3_Si_6_) phase and the Mg_2_Si phase are formed at the last stage of solidification; the incomplete modified eutectic silicon is near the π(Al_8_FeMg_3_Si_6_) phase and Mg_2_Si phase. The eutectic silicon phase in the core of eutectic structure was fully modified, and the partially modified eutectic silicon only existed at the edge of the eutectic structure. Therefore, it can be believed that coarse eutectic silicon was formed at the last stage of solidification. During solidification, alloying element Mg will be rejected by both eytectic constituents during the melting. The segregation of Mg results in the formation of a constitutionally supercooled zone, which changes the morphology of the eutectic silicon phase. The accumulation of Mg atoms ahead of the eutectic silicon phase causes a shift of the melting point down extensions of the liquidus surface, resulting in a decrease in freezing rate leading to coarsening of the eutectic silicon [23]. The addition of Mg in Al-Si hypoeutectic alloy resulted in a decrease in the eutectic nucleation frequency; the constitutional driving force for growth began to decrease; the interface velocity slowed down to a velocity where the growth mode of eutectic silicon morphology changes [19]. As mentioned above, the alloying element Mg affects the eutectic nucleation and growth dynamics in Al-7Si-0.4Mg alloy at the last stage of solidification. Thus, it can be observed that the partially-modified eutectic silicon has an adhering relationship with the π(Al_8_FeMg_3_Si_6_) phase and Mg_2_Si phase, which formed at the last stage of solidification.

## 4. Conclusions

(1) The alloying element Mg has an enhanced effect on the uptake of sodium in the Al-Si hypoeutectic alloy modified by sodium-contained salt. With the same addition rate of sodium-contained salt modifier, the sodium content in Al-7Si-0.4Mg was found to be significantly higher than that in Al-7Si alloy. The initial sodium content of commercial pure Al-7Si and Al-7Si-0.4Mg alloy is 1.3 ppm and 2.2 ppm, respectively. After adding 0.2 wt.%, 0.4 wt.%, 0.6 wt.% of Na-based modifier, residual sodium levels increased to 14.6 ppm, 31.1 ppm, 46.3 ppm and 33.3 ppm, 71.7 ppm, 85.4 ppm, respectively.

(2) When 0.6 wt.% Na-contained salt modifier or 0.03 wt.% Sr was added, the eutectic silicon of Al-7Si alloy underwent the desired modification. With the addition of 0.2 wt.% Na-contained modifier or 0.03 wt.% Sr, the eutectic silicon morphology was fibrous at the core of the eutectic structure; some coarse eutectic silicon could be observed at the edge of the eutectic structure of the Al-7Si-0.4Mg alloy. The incomplete modification of eutectic silicon could not be eliminated by increasing the addition level of modifier or by decreasing the Fe content in the alloy.

(3) The modification of eutectic silicon in Al-7Si-0.4Mg alloys was degraded by the addition of Mg. For both Na modification and Sr modification, the eutectic silicon exhibited incomplete modification in the Al-7Si-0.4Mg alloy. Moreover, there was an adhering relationship between the partially modified eutectic silicon with Mg-rich phases. The partially modified eutectic silicon formed at the last stage of solidification was probably caused by the change of solidification conditions of the alloy due to the presence of alloying element Mg.

## Figures and Tables

**Table 1:** The chemical composition (wt.%) of alloys.

Elements	Si	Mg	Na	Sr	Fe	P	Al
Commercial pure Al-7Si	6.95752	0.00041	0.00013	0.00021	0.09012	0.00117	Balance
Commercial pure Al-7Si-0.4Mg	7.05375	0.39372	0.00022	0.00019	0.10482	0.00122	Balance
High pure Al-7Si	7.26770	0.00034	0.00008	0.00021	0.00915	0.00081	Balance
High pure Al-7Si-0.4Mg	7.17539	0.39532	0.00010	0.00017	0.01093	0.00072	Balance

## Data Availability

The data presented in this study are available on reasonable request from the corresponding author. The data are not publicly available due to privacy.
